# Gastro-Entero-Colitis of Infancy and Childhood

**Published:** 1892-11

**Authors:** G. W. Christian

**Affiliations:** Burnet, President Austin District Medical Society


DANIEL'S TEXAS MEDICAL JOURNAL.

ESTABLISHED JULY, 1885.

PUBLISHED MONTHLY.—SUBSCRIPTION $2.00 A YEAR.

Vol. VIII. AUSTIN, NOVEMBER, 1892. No. 5.

### Original Contributions.

For Daniel's Texas Medical Journal.

### GASTRO-ENTERO-COLITIS OF INFANCY AND CHILDHOOD.

G. W. CHRISTIAN, M. D., BURNET, PRESIDENT AUSTIN DISTRICT MEDICAL SOCIETY.

[Read at Waco, Texas, October 11, 1892, at joint session of Texas Central District and Austin District Medical Societies.]

FOR the use of this term in discussing the diarrhœal diseases of infancy and childhood, I have no other apology than the fact that they all often exist in the same individual, and there are no unfailing symptoms by which we can differentiate; hence, in the study of this class of troubles, I feel that we are more than warranted in the use of the compound term which, as far as I am able to judge, has generally been used only in relation to cholera infantum. I shall use the term as expressive of all forms of inflammatory diarrhœa and dysenteric diseases. I do not regard cholera infantum as a separate and specific disease, but simply an exaggerated form of gastro-intestinal inflammation, and shall speak of the disease in its milder and its malignant forms as one and the same. The importance of a thorough understanding of these classes of disease is fully appreciated by the medical profession, when we see day after day the little joys of the household falling one by one into a premature grave; when we hear the disconsolate mothers' sobs, and look at their anxious, beseeching faces, pleading, "Oh, Doctor, can't you do something to save my baby?" we are forcibly reminded of the sacredness of the trust reposed in us, and for my part, if I do no more to-day than stimulate each of you to a more thorough study of this subject than you have heretofore given it, I shall feel that I have not labored in vain. I have for several months (since reading an appeal in DANIEL'S TEXAS MEDICAL JOURNAL, from a physician to the profession, asking for aid, saying that not only his own three little ones had gone from home to the grave, but that patient after patient had gone the same way, and in spite, too, of the fact that he had called upon the best accessible counsel, and had exhausted almost, the pharmacopea, to no avail), wanted to write upon this subject, but a busy country work has caused neglect until now. We are told that half of the children born, die before they reach the fifth year; then, that half, or more, die of gastro-intestinal diseases, stamps it at once as the most important that we are called upon to treat.

*Predisposing Causes.*—The disease is not so frequent, nor so fatal, in the country as in the cities, but wherever children are born and reared, the disease will be found, whether upon the lowlands or mountain peaks, whether upon the bosom of the lowland swamp or of the dry and arid plains. It exists, too, every month in the year; but the following table, from New York Board of Health, 1882, indicates clearly that season has more to do with it than any other one factor: Deaths, under five years of age, January, 34; February, 32; March, 50; April, 50; May, 72; June, 231; July, 1533; August, 817; September, 362; October, 195; November, 68; December, 35. Thus it will be observed that nearly twice as many deaths occurred in July as in nine other spring, fall, and winter months. Next to heat is foul air and filth, want of cleanliness in the person of the child, —being confined in a close, hot, dirty room would prostrate even the more mature subject. That these elements are of much more importance than the above table would on its mere face indicate, we have only to remember that the disease is not near so common in the country, where the degree of heat obtains, but minus the poisonous gases common to a dense population, in the absence of rigid cleanliness. Then it appears that a high temperature, conjoined with filth, are the principal factors in the production of this disease. Whether it is because a miasm is generated by these factors, we are not sure, but that they are most potent factors as predisposing causes, we have abundant proof. What part germs play in the production of this disease, we are still somewhat at sea, but all facts point strongly to the fact that they have more than a coincidental relation thereto. Antiseptic surgery has taught us that we can have wounds without suppuration and other evidences of inflammation, then it would be an easy and natural inference that we would and could have gastro-intestinal inflammation only from the presence of bacteria. If wounds from cuts and irritants on the outside of the body heal, under proper antiseptic treatment, without inflammatory action, then we would naturally expect that the same would follow like treatment inside the body, if we could as thoroughly render antiseptic such parts.

*Exciting Causes.*—Taking cold from exposure and improper and insufficient clothing has some influence in precipitating an attack. Dentition is another exciting cause of no mean importance, though, I believe, generally much exagerated as a causative factor, and more especially among the laity. Feeding I regard as the most common and important of the exciting causes; this may follow from the character of the food itself, or from being given in too great quantities. The bottle-fed infants are more than twice as liable to be affected than the wet-nursed, and the mixed-fed, while not so apt to take the disease as the pure bottle-fed, are still much more liable than the strictly wet-nursed.

*Symptoms.*—In mild cases the daily discharges gradually increase from one or two to half a dozen or more, the child grows restless and fretful, becomes feverish, and craves the breast, or bottle, oftener than usual. The discharges gradually increase in number, and the child begins to suffer with tormina as the stools are passed; the actions change in character from time to time, sometimes mattery or watery, greenish yellow to brown, at others lumpy, with particles of mucus or blood, or both, and as the trouble progresses the actions will contain bloody matter, either alone or mixed with thin watery fluid, and lumps of indigested and partly decomposed food; the child continues to grow more restless and feverish as the disease advances, and if not relieved in a few days, then symptoms are all exaggerated, vomiting is generally added to the already distressing condition, and the child rapidly wastes away, until its features are sunken, the bony prominence stand out prominently; its skin lays in loose folds on its extremities; it throws its hands and feet about, and its head, and sometimes its body, from side to side; its countenance grows dull; its eyes lose their brightness, and the round, plump, smiling baby of a few days ago succumbs to its suffering and passes, a dried up skeleton, to the grave. Sometimes vomiting is the first symptom, the diarrhœal trouble following rapidly in its wake, at other times the stomach remains quiet throughout the attack, and tolerates any amount of nauseous drugs that may be forced upon it. In the majority of cases vomiting begins about the 6th or 8th day of the illness; this is often the first symptom to alarm the mother, as many people believe that diarrhœa is one of nature's methods of relieving teething children, and should be left alone.

*Treatment.*—The child should, as soon as taken, be placed in a cool, clean, light, and well ventilated room; its clothing should be light (if in the hot months), and kept scrupulously clean, the patient should have daily, tepid baths, and if much fever, frequent cold sponging, and if very bad, no food at all should be given for 6 or 8 hours, and then only such as raw white of an egg with water, or the liquid, predigested foods, for 24 to 40 hours longer, owing to the severity of the case; it makes no difference whether the child is fed or wet-nursed, all diet, and especially all milk diet, should be withheld for a few hours at least. Wash the stomach and bowels out with water; if vomiting is frequent, the child will do this itself, if you give freely all the water it will drink. The water cools its fever, and as vomited back, brings the irritating matter with it; cleanses its stomach out as it were. If, after a calomel purge, the actions continue lumpy, bloody, or mattery, irrigate the lower bowels with hot or cold water, plain or medicated, to suit the case, by passing a catheter, attached to a fountain syringe. Do this once, twice, thrice a day. Small doses of calomel, repeated every two to six hours for the first 36 hours of the treatment, have given me the best and most satisfactory results obtained by drugs. Give food of any kind sparingly for 24 hours, and then do not feed beyond the digestive capacity. In returning to the bottle or breast, see that the foods themselves are not at fault. I might mention the whole list of drugs found useful in this disease, but you are all familiar with them, and I have probably said enough to bring the matter before you; and after emphasizing my own idea of the proper methods in the earlier stages of the disease, which is, empty the child's stomach of food and fecal matter, and give a few hours rest from food of any kind (giving, though, plenty of water), and feed for 24 to 48 hours, on foods that will not coagulate, or leave lumpy residue in the gastro-intestinal tract.

I will now leave the subject to abler hands.*

*We regret being unable to get a report of the able discussion which followed this paper, the JOURNAL having no representative preseni.—ED.

---

For Daniel's Texas Medical Journal.

